# Identification of an atypical microRNA with sequence variation in the silkworm *Bombyx mori*

**DOI:** 10.1371/journal.pone.0346656

**Published:** 2026-10-06

**Authors:** Mayuko Harada, Midori Tabara, Kazunori Kuriyama, Katsuhiko Ito, Hidemasa Bono, Takuma Sakamoto, Miho Nakano, Toshiyuki Fukuhara, Atsushi Toyoda, Asao Fujiyama, Hiroko Tabunoki

**Affiliations:** 1 Department of Science of Biological Production, Graduate School of Agriculture, Tokyo University of Agriculture and Technology, Fuchu, Tokyo, Japan; 2 Ritsumeikan-Global Innovation Research Organization, Ritsumeikan University, Kusatsu, Shiga, Japan; 3 Department of Applied Biological Sciences, Tokyo University of Agriculture and Technology, Fuchu, Tokyo, Japan; 4 Database Center for Life Science (DBCLS), Joint Support-Center for Data Science Research, Research Organization of Information and Systems (ROIS), Mishima, Shizuoka, Japan; 5 Laboratory of Bio-DX, Genome Editing Innovation Center, Hiroshima University, Higashi-Hiroshima City, Hiroshima, Japan; 6 Laboratory of Genome Informatics, Graduate School of Integrated Sciences for Life, Hiroshima University, Higashi-Hiroshima City, Hiroshima, Japan; 7 Advanced Genomics Center, National Institute of Genetics, Mishima, Shizuoka, Japan; 8 Cooperative Major in Advanced Health Science, Graduate School of Bio-Applications and System Engineering, Tokyo University of Agriculture and Technology, Fuchu, Tokyo, Japan; Kyushu University Faculty of Agriculture Graduate School of Bioresource and Bioenvironmental Sciences: Kyushu Daigaku Nogakubu Daigakuin Seibutsu Shigen Kankyo Kagakufu, JAPAN

## Abstract

MicroRNAs (miRNAs) play essential roles in posttranscriptional regulation of gene expression. During miRNA biosynthesis, miRNA precursors are cleaved by Dicer at their loop structure to generate mature miRNAs. However, the functional consequences of sequence alterations in the loop region remain poorly understood. The silkworm *Bombyx mori* is a lepidopteran insect with many genetic strains. In a *B. mori* strain exhibiting translucent larval skin, we unexpectedly identified a mutant form of *miR-3260* in which part of the predicted loop structure was deleted. Here, we aimed to analyze the function of wild-type *miR-3260* and the impact of this loop structure mutation in *B. mori.* First, we predicted potential binding partners for wild-type *miR-3260* using the RNAhybrid tool and identified eight candidates, including two juvenile hormone (JH)-related genes. Expression analysis revealed high *miR-3260* expression in the corpora allata. Furthermore, treatment of BmN cells with JH led to upregulated *miR-3260* expression, suggesting a relationship between *miR-3260* and JH signaling. Although *miR-3260* mimics and inhibitors altered the expression of two JH-related candidate genes, the observed changes were not consistent with the predicted regulatory effects of *miR-3260*, and no observable larval phenotype was detected. Additionally, analysis of Dicer processing showed that neither the wild-type nor mutant *miR-3260* precursor underwent Dicer-mediated cleavage. Although *miR-3260* responds to JH, its function as a conventional miRNA was not supported. These findings suggest that *miR-3260* may act as a JH-responsive non-coding RNA or represent a novel atypical RNA distinct from canonical miRNAs.

## Introduction

Silkworm *Bombyx mori* is a lepidopteran insect long used to produce silk fiber. Advances in genetic research have facilitated breeding of silkworms that can produce high-quality silk, resist disease, and construct large cocoons; silkworm strains with favorable traits have been collected from around the world [1]. Various interesting phenotypes have been maintained as a genetic resource for future use [1] and linked to causative genes via linkage maps. Currently, 561 strains are maintained at the National Bioresource Project (NBRP) KAIKO [2].

In addition, *B. mori* has been used as a lepidopteran model insect in genetic, physiological, and pharmacological research [3]. Its genome sequence has been reported [4], as has the role of microRNAs (miRNAs) in gene expression regulation in this organism [5,6].

miRNAs are short (18–25 bases), untranslated, single-stranded RNAs that play important roles in the posttranscriptional regulation of gene expression [7,8]. They bind to the 3’ untranslated region (3’UTR) of target gene mRNAs with incomplete complementarity and suppress translation of the target gene [9]. miRNAs form a stem‒loop precursor when transcribed in the nucleus [10], being then cleaved by Dicer in the cytoplasm [9,11], followed by several modifications to become mature miRNAs [12]. The 2–8 base sequence at the 5’ end of mature miRNAs is called the seed sequence, which binds to the target mRNA with perfect complementarity [13,14]. Each miRNA can target different mRNAs, which are degraded to control gene expression [9]. Dicer recognizes the nucleotide (nt) sequence and secondary structure of the miRNA precursor at the cleavage site [15–19]. miRNA precursors have a loop structure [20, 21], which does not exist in mature miRNAs [22–24]. However, it remains unclear whether the absence of a correct nucleotide sequence in the loop structure is necessary for Dicer recognition [19,23,24].

Therefore, miRNAs posttranscriptionally regulate processes, such as metabolism, embryonic development, differentiation, disease response, and immunity, across organisms [12–14,25,26]. As of 2024, 38,589 hairpin precursors and 48,860 mature miRNAs from 271 organisms have been registered and published in the miRbase database [27].

We happen to identified a mutant form of *miR-3260* lacking part of the predicted loop structure in a silkworm mutant strain, o-751 (*op* mutant), which displays spontaneous translucency during the larval stage, high pupal-stage mortality, and male infertility. The *op* phenotype is caused by mutations in the *Bm-cN-II* gene [28]; however, during positional cloning of the *op* responsive region, we incidentally discovered m*miR-3260* (Fig 1). Here, we aimed to analyze the role of wild-type *miR-3260* and the influence of the mutation of the loop structure in gene expression in *B. mori.*

**Fig 1:**
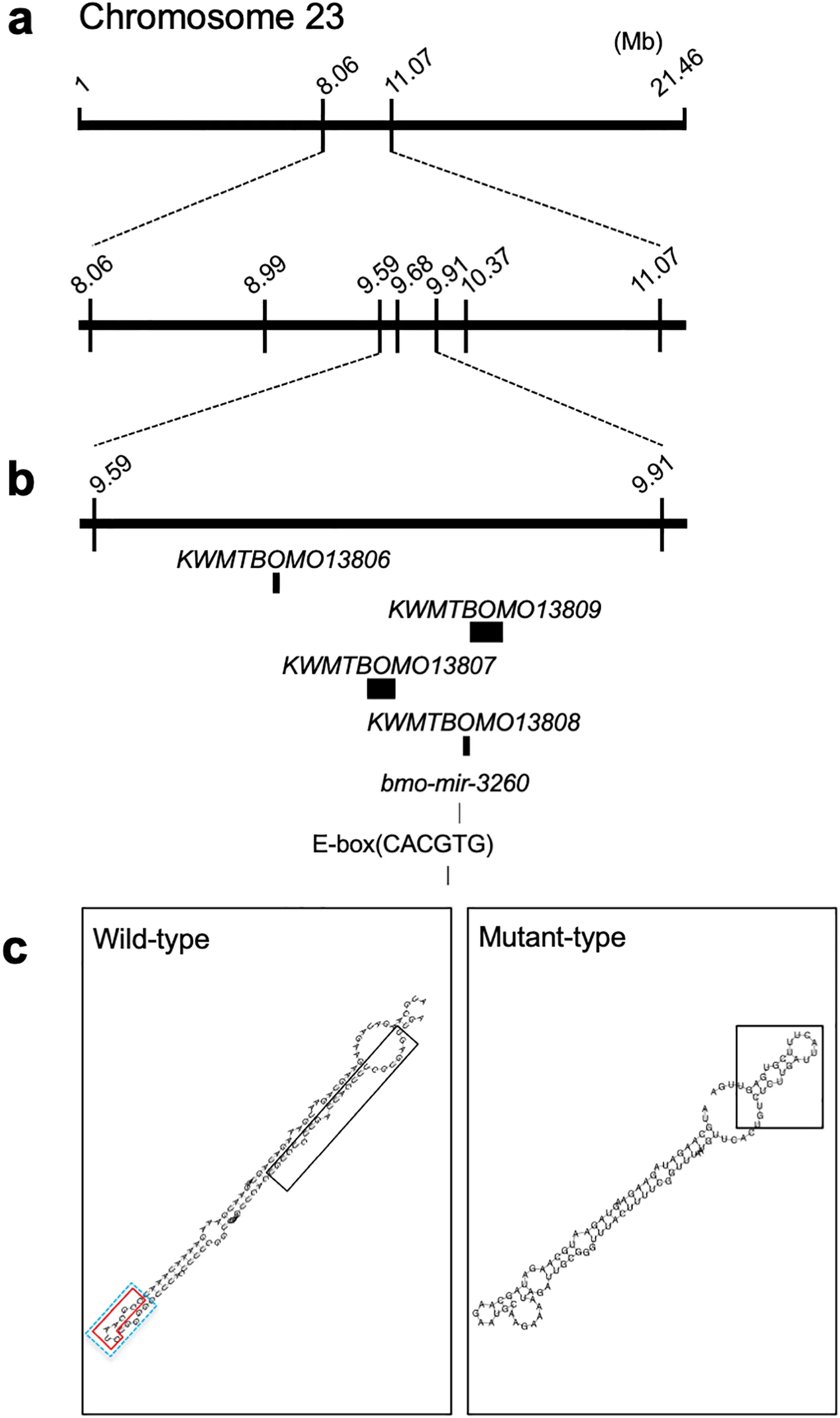
Identification of a *miR-3260* variant on chromosome 23. (a) Positional cloning of chromosome 23. Each number indicates the chromosomal position of the marker listed in S1 Table in S1 File. The *op* locus was located within a region ~0.32 Mb in length between 9,598,832 and 9,916,751 bp on chromosome 23. This mapped region is distinct from the previously reported *Bm-cN-II* locus [28]. (b) Predicted genes within the region, including *KWMTBOMO13806*, *KWMTBOMO13807*, *KWMTBOMO13808*, and *KWMTBOMO13809*, and one miRNA, namely, *bmo-mir-3260* (MIMAT0015444) with E-box. (c) Prediction of the miRNA secondary structure. The secondary structure was predicted based on the *miR-326*0 nucleotide sequence using RNAfold WebServer. Wild-type indicates wild-type *mir-3260* in the p50 silkworm strain, and mutant indicates m*mir-3260* in the o-751 silkworm strain. The black box shows the mature sequence region, a blue dashed box indicates the predicted loop structure, and the red box missing nucleotide sequences in the *miR-3260* mutant.

## Materials and methods

### Silkworm strains

Wild-type (+^*op*^/ + ^*op*^) and the *op* mutant (*op*/*op*) o-751 strains were supplied by Kyushu University in the National Bio Resource project. C108 and p50 strains were maintained by Tokyo University of Agriculture and Technology. For positional cloning, F_2_ progeny from the cross F_1_ (o75 × C108) female × F_1_ (o75 × C108) male were used (S1 Table in S1 File). All silkworm larvae were reared on mulberry leaves at 25–28ºC. The *B. mori* strain N4 used in this study was supplied by the University of Tokyo. Silkworm larvae were reared on the artificial diet silk mate 2S (Nosan, Tsukuba, Japan). Insects were maintained at 25ºC under a 16-h light/8-h dark cycle.

### Cell culture

A silkworm cell line, BmN (RCB0457), was obtained from the Riken Cell Bank (Riken Tsukuba, Japan). BmN cells were maintained at 25ºC in TC-100 medium (Thermo Fisher, Waltham, Massachusetts, USA) supplemented with 10% fetal bovine serum and an antibiotic-antimycotic mixture (Invitrogen, Carlsbad, CA).

### Identification of *miR-3260* via positional cloning

For positional cloning, SNP and PCR markers were generated at various positions on chromosome 23, and polymorphic markers between the parents were used for linkage analysis of 259 F_2_ larvae. The primer sequences used in this study are shown in S2 Table in S1 File. Candidate genes in the region narrowed by positional cloning were annotated using Silkbase [29].

### Preparation of genomic DNA and polymerase chain reaction (PCR) analysis

Genomic DNA was prepared using silk glands from 5^th^-instar larvae of the o-751 strain, employing DNAzol Reagent (Cosmo Bio) according to the manufacturer’s protocol. PCR was performed with Ex Taq DNA Polymerase (Takara Bio) using the primer sets listed in S2 Table in S1 File, designed using the novel *Bombyx* draft genome sequence [30]. PCR conditions were as follows: initial denaturation at 94ºC for 2 min; 40 cycles of denaturation at 94ºC for 15 s, annealing at 60ºC for 15 s, and extension at 72ºC for 1 min; and a final incubation step at 72ºC for 4 min.

### Verification of the miRNA mutation

Genomic DNA was extracted from the silk glands of either wild-type or *op* mutant silkworms from the o-751 strain (n = 2 each) using a QIAprep Spin Miniprep Kit (QIAGEN, Venlo, Netherlands). For paired-end library preparation, the extracted genomic DNA was cut to an average size of 400 bp using a Covaris Acoustic Solubilizer S220 (Covaris Inc., MA. USA). Sequencing libraries were constructed using the TruSeq DNA PCR-Free Sample Prep Kit (Illumina, CA, USA) according to manufacturer’s instructions and sequenced on the HiSeq 2500 system (Illumina, CA, USA) using a read length of 2 × 150 bp. Based on the locus identified by positional cloning, we aimed to differentiate the nucleotide sequences from those of the standard strain p50. Next, we examined the target gene or miRNA, including differentiation at the locus utilizing RNA-seq data processed with HISAT2 (v2.0.1-beta) [31] and Stringtie (v1.2.2) [32]. *B. mori* miRNA data were obtained from miRbase [27]. Using the ultrafast sequence search tool GGGenome [33], those sequences were remapped to the genome assembly (NCBI accession no. GCF_000151625.1), and the promoter region was checked. Only one miRNA (*bmo-mir-3260*) was mapped to the locus identified by positional cloning.

### Purification of miRNAs and total RNA from silkworm tissue

Whole body samples of the N4 *B. mori* strain were collected and pooled for each developmental stage: eggs (n = 10), 1st-instar larvae (1L, n = 3), 2nd-instar larvae (2L, n = 3), 3rd-instar larvae (3L, n = 3), 4th-instar larvae (4L, n = 3), 5th-instar larvae (5L, n = 3), and pupae (n = 3). The corpora allata was dissected from day-4 5th-instar larvae of the N4 *B. mori* strain (n = 10) and pooled into a single sample. Each sample was treated with QIAzol Lysis Reagent from the miRNeasy Mini Kit (QIAGEN) or lysis buffer from the Pure Link RNA Mini Kit (Life Technologies) and stored at −80 ºC until use. miRNA was purified using the miRNeasy Mini Kit (QIAGEN) and RNeasy MinElute Cleanup Kit (QIAGEN) according to manufacturer’s instructions. Total RNA was purified via a Pure Link RNA Mini Kit (Life Technologies) according to manufacturer’s instructions.

### cDNA library synthesis using purified miRNA and mRNA

A total of 200 μg of purified miRNA from each sample was used to synthesize the cDNA library with the TaqMan™ MicroRNA Reverse Transcription Kit (Thermo Fisher, Waltham, Massachusetts, USA). cDNA libraries were stored at −30 ºC until use. One microgram of purified total RNA was treated with DNase I (Thermo Fisher) and cDNA synthesized using the PrimeScript^TM^ 1st strand cDNA Synthesis Kit (Takara Bio Co. Ltd., Tokyo Japan) according to manufacturer’s instructions.

### Confirmation of the miRNA sequence

To confirm the *miR-3260* sequence, DNA purified from the silk glands of 4-day-old 5th-instar larvae of *B. mori* o-751 and the p50 strain (n = 2 each) was used as template to amplify the miRNA sequence using KOD Plus Neo (TOYOBO, Tokyo, Japan), and the primer pair 5’-ATGCAAGATAGAAGAAGTAG-3’ and 5’-TCAACTCACGAAAGTAATC-3’ was designed based on the genome sequence. The amplified PCR products were electrophoresed on a 1% agarose gel, and bands of the target nucleotide length were excised and extracted using the QIAquick Gel Extraction Kit (50) (QIAGEN, Venlo, Netherlands). 3’A was added to both ends of extracted PCR products using 10 × A-attachement Mix (Toyobo Co., Ltd., Tokyo, Japan) and ligated into the pCR 2.1-TOPO vector (Life Technologies, Waltham, Massachusetts, USA). The vector with the ligated miRNA sequence was transformed into ECOS^TM^ Competent *E. coli* XL1-Blue (NIPPON GENE, Tokyo, Japan). Transformed *E. coli* cells were then inoculated onto LB agar medium supplemented with 100 μg/mL ampicillin and incubated at 37 ºC for 16 h. Then, colonies were examined by direct colony PCR with M13 primers (S3 Table in S1 File) to determine whether the target *bmo-mir-3260* sequence was present. The clone of interest was cultured under shaking at 37 ºC for 16 h in liquid LB medium supplemented with 100 μg/mL ampicillin. Then, the culture was centrifuged at 3,500 rpm for 20 min to collect bacterial cells and the plasmid DNA purified using a QIAprep Spin Miniprep Kit (QIAGEN, Venlo, Netherlands). The nucleotide sequence of the resulting plasmid DNA was confirmed by the Sanger method using a 3730xl DNA analyzer (Life Technologies, Waltham, Massachusetts, USA).

### Quantitative reverse transcription PCR for miRNA

A volume of 2 μL of cDNA was used as template. TaqMan™ Fast Advanced Master Mix (Thermo Fisher, Waltham, Massachusetts, USA) and *miR-3260* primers (Thermo Fisher, TaqMan miRNA Assay, Assay ID: 241990_mat) were mixed with each miRNA cDNA library, and quantitative RT‒PCR was performed using the Step One Plus Real‒Time PCR system (Applied Biosystems) according to manufacturer’s instructions. Relative miRNA expression levels were calculated using the delta‒delta Ct method based on the Ct values obtained by quantitative RT‒PCR. U6 (Thermo Fisher, TaqMan miRNA Assay, Assay ID: 001973) was used as an endogenous control. miRNA expression levels in whole bodies are shown as RQ values. Whole-body miRNA expression was normalized to 1st-instar larvae (RQ = 1), whereas corpora allata expression was normalized to 5th-instar, whole-body larval samples (RQ = 1). Error bars indicate the RQ minimum and maximum values based on standard deviation. All assays were performed in technical triplicate.

### Quantitative reverse transcription PCR for total RNA

A volume of 2 μL of cDNA was used as template. RT-qPCR was performed on cDNA libraries synthesized from total RNA using primers for each gene and the KAPA SYBR Fast qPCR Kit (NIPPON Genetics) according to manufacturer’s instructions. The relative mRNA expression level for each gene was calculated using the delta‒delta Ct method based on the Ct values obtained by quantitative RT‒PCR. Ribosomal protein 49 (Rp49) was used as an endogenous control (S3 Table in S1 File). mRNA expression levels in each sample are shown as RQ values, normalized to control samples (RQ = 1). Error bars reflect RQ minimum and maximum levels based on standard deviation. All assays were performed in triplicate.

### Prediction of miRNA secondary structure and target mRNA binding

Secondary structures based on wild-type or mutant *miR-3260* nucleotide sequences were predicted using RNAfold WebServer [34]. We searched for mRNA sequences that bind to miRNAs using transcriptomic data from the standard silkworm strain p50T and the mutant strain o-751 (PRJDB19213). The method for RNA-seq analysis is detailed in the Supporting Information. First, we identified homologous miRNA sequences in miRbase, confirmed their mature sequences, and then obtained seed sequences. According to the silkworm gene database, *miR-3260* is located on chromosome 23, adjacent to cluster of JH-related genes (Chr23; 9916751−10377814). Since coordinated expression of neighboring genes has been documented in various species [35], we speculated that *miR-3260* expression may be involved in regulating nearby JH-related genes. To test this hypothesis, we retrieved data on the relevant gene clusters from KAIKObase [36]. Next, we extracted the 3′UTR sequence from the transcriptome data of the fat body of larvae from the o-751 strain on the 3rd day of the 5th instar. Using these data and the JH-related gene cluster sequence, we used RNAhybrid [37] to search for mRNAs with sequences complementary to the seed sequence. As conditions for binding to the target sequence, the minimum free energy (mfe), which indicates the ease of binding, was set to ≤ −20 [38], and the seed sequence could have a G:U wobble.

### Examination of *miR-3260* expression under JH treatment

BmN cells (2 × 10^6^) were grown on 6-well Falcon plates (BD Biosciences, Franklin Lakes, NJ). JH III was purchased from Cayman Chemical (Michigan, USA) and dissolved in 10% isopropanol to prepare a 10-mg/mL stock solution. The stock solution was diluted with 1x insect PBS (10 mM phosphate and 150 mM NaCl; pH 6.5) to 100 μg/mL. In total, 50 μL of the diluted JH solution was added to each well containing BmN cells (n = 3), yielding a final JH concentration of 9.4 μM per well. For controls, a 10% isopropanol solution was diluted 1:100 with 1x insect PBS, and 50 μL of this solution was added to each well with BmN cells (n = 3). JH-treated BmN cells were cultured at 25 ºC for 24 and 48 h, harvested, pooled by treatment group, and subjected to miRNA purification and RT-qPCR.

### Injection of *miR-3260* mimic or inhibitor into *B. mori* eggs

*B. mori* N4 strain adults were mated and their eggs used for injection within 4 h after laying. Using an electric manipulator and a microinjector, eggs were perforated with a tungsten needle so that egg contents leaked onto the eggshell surface. Then, 5 nL of the 100 μM of *miR-3260* mimic (Thermo Fisher, product ID: MC18121, S4 Table in S1 File) or inhibitor (Thermo Fisher, product ID: MH18121, S4 Table in S1 File) was injected into the egg liquid with a glass capillary; we confirmed that the egg liquid returned to the inside of the egg before sealing the hole with instant adhesive. Water was used as control. Eggs were incubated at 25 ºC until hatching (n = 48) or until reaching the 2nd-instar larval stage (n = 5). Whole-body samples of 2nd-instar larvae were used for *JHAMT* and *JHBP* mRNA expression analysis (S3 Table in S1 File).

### Dicing assay

Dicing (Dicer) assays were performed according to Tabara et al. [39]. Cultured BmN cells in T25 flasks and dissected fat bodies from the N4 *B. mori* strain were sonicated on ice in extraction buffer (20 mM Tris-HCl [pH 7.5], containing 4 mM MgCl_2_, 5 mM DTT, 1 mM PMSF, 1 μg/mL leupeptin, and 1 μg/mL pepstatin A). BmN cell and fat body lysates were centrifuged twice at 13,000 × *g* and 4 ºC for 15 min and supernatants collected with protein concentrations adjusted to 0.1 mg/mL. Subsequently, 1 μL of ^32^P (0.37 MBq/µL) end-labeled dsRNA or miRNA precursor single-stranded RNA (ssRNA) (*pre-bmo-let7, pre-bmo-*m*let7, pre-miR-3260*, and *pre-*m*miR-3260*; Agilent Technologies, Palo Alto, CA, USA; S5 Table in S1 File) was incubated with 15 μL of BmN or fat body lysate containing BmDicer-1 and BmDicer-2 proteins and 4 μL of 5 × dicing buffer (18 mM HEPES-KOH [pH 7.4], with 7.5 mM DTT, 3.3 mM magnesium acetate, 100 mM potassium acetate, 0.25% glycerol, and 1 mM ATP) at 25 ºC for 30 min. After incubation, fractions were separated via 15% denaturing PAGE and analyzed using autoradiography. After 24 h, cleaved RNAs were visualized as band intensities using a Typhoon FLA 7000 image analyzer and ImageQuant TL (GE Healthcare Japan).

### Statistical analysis

Statistical significance was assessed in R (ver 4.3.1) using Fisher’s exact test with multiple comparisons. Holm’s method is used to correct the familywise error rate. *P* < 0.05 was considered statistically significant.

## Results

### Identification of a *miR-3260* variant during analysis of the *op* chromosomal region

The *B. mori* linkage map locates the *op* phenotype’s causative gene on chromosome 23 [29], with *Bm-cN-II* (KWMTBOMO13770) previously identified as the causative gene [28]. During positional cloning analyses of chromosome 23, we unexpectedly identified an *miR-3260* variant in a region separate from the *Bm-cN-II* locus. Concretely, by genotyping 259 F_2_ individuals, we delimited the locus to a ~ 320-kb region on chromosome 23 (Fig 1a and S1 Table in S1 File). This interval differs from the location of *Bm-cN-II*, which was previously reported as the causative gene of the *op* phenotype [28]. SilkBase [29] revealed that this region contained four predicted genes, namely, *KWMTBOMO13806*, *KWMTBOMO13807*, *KWMTBOMO13808*, and *KWMTBOMO13809*, and one miRNA, namely, *bmo-mir-3260* (Fig 1b). We subsequently compared the sequences of the region containing these genes with the genome sequence of the standard strain p50. We identified a *bmo-mir-3260* mutant lacking an 8-nt sequence in the o-751 strain silkworm; the deletion was located in a predicted loop structure in *bmo-mir-3260* (Fig 1c). Additionally, we checked upstream of the *miR-3260* nucleotide sequence, and found JH response element (JHRE) sequences (cacgtg) [40] at Bomo_Chr23: 9795152–9795269 (Fig 1b). We called the wild-type *bmo-mir-3260* in the p50 silkworm strain as *miR-3260* and the *bmo-mir-3260* lacking the predicted loop structure as m*miR-3260* in the o-751 silkworm strain.

### Determination of the *mir-3260* sequence

To determine the nucleotide sequence of *miR-3260*, we amplified the target miRNA via PCR using DNA extracted from the silk glands of p50 or o-751 strains (+/+ or *op*/*op*, respectively) as templates. The 110-base sequences were obtained from the o-751 strain. We then confirmed the presence of an m*miR-3260* sequence in op/op, + /op, and +/ + o-751 strains; it was conserved in the *op*/*op*, + /*op*, and +/ + o-751 strains but not in the p50 strain. Sequence analysis revealed that m*miR-3260* is eight nucleotides shorter than wild-type *miR-3260* and differs at five nucleotide positions (S1 Fig in S1 File). The m*miR-3260* nucleotide sequence was submitted to the International Nucleotide Sequence Database (DNA Data Bank of Japan, DDBJ) under accession number LC858578.

### Prediction of binding potential to *miR-3260*

Using the *B. mori* o-751 strain fat body transcriptome data, we searched for target mRNAs that may bind to *miR-3260* (Fig 2). Genes located close together on the same chromosome often exhibit coordinated expression, as demonstrated in humans, mice, rats, yeast, fruit flies, and worms [35]. Therefore, we also checked genes close together on the same chromosome using KAIKObase. The region near *miR-3260* (Chr23; 9916751–10377814) contained a JH-related gene cluster, and *miR-3260* had JHRE. We speculated that *miR-3260* expression may be linked to the expression of these JH-related genes. Thus, we focused on the JH-related gene cluster close to *miR-3260* on Chr23 (9916751–10377814).

**Fig 2:**
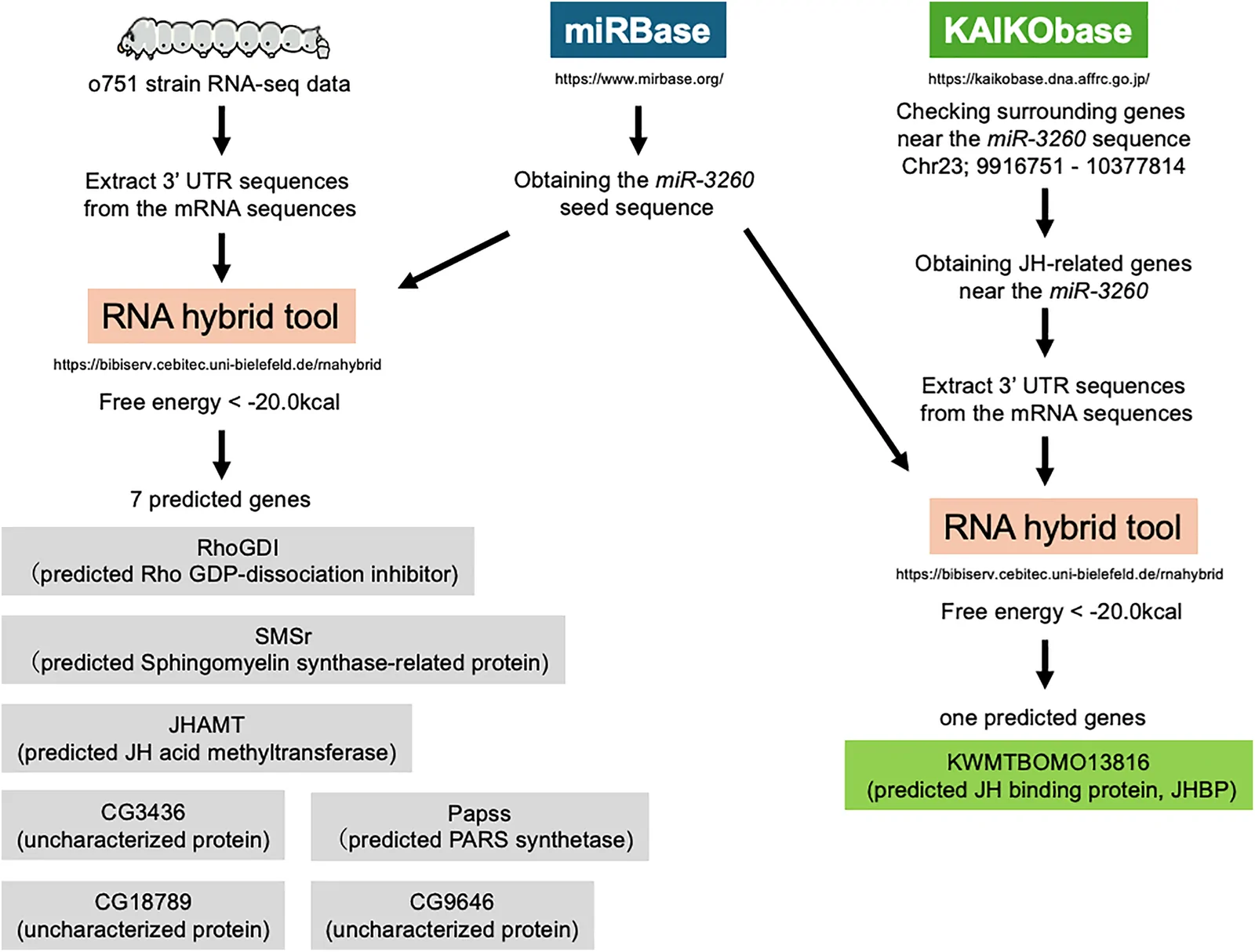
Prediction of binding potential to *miR-3260.* mRNA sequences that may bind to *miR-3260* were predicted using RNAhybrid. The mfe value was set to ≤ −20 and the seed sequence allowed to have a G:U wobble. The image of the silkworm, translucent larval skin mutant, was extracted from TogoTV (© 2016 DBCLS TogoTV, CC-BY-4.0 https://creativecommons.org/licenses/by/4.0/deed.ja).

We obtained 3’UTR sequences of JH-related genes near *miR-3260* from KAIKObase [36] and 3’UTR sequences from our RNA-seq data and then analyzed their binding potential using the RNAhybrid tool [37]. As a result, we found seven candidate genes binding to *miR-3260* using *D. melanogaster* gene annotations in RNA-seq data: RhoGDI (Rho GDP-dissociation inhibitor), CG3436, CG18789, PAPS synthetase (PARS synthetase), CG9646, SMSr (Sphingomyelin synthase-related protein), and juvenile hormone acid methyltransferase (*JHAMT*). Furthermore, in KAIKObase, we found a gene near *miR-3260*: *B. mori* juvenile hormone binding protein (*JHBP*) ce-0330 (*KWMTBOMO13816*, AB196703), a member of the JH-related gene cluster (Fig 2).

### *miR-3260* expression at various developmental stages and in the corpora allata

We analyzed *miR-3260* expression from embryonic to pupal stages (Fig 3a). Compared with the 1st-instar larval stage, *miR-3260* expression levels were higher in embryos at 216 h after oviposition and in 1st-instar larvae. In a breeding environment at 25 ºC, eggs hatch in ~10 days (240 h). *miR-3260* expression gradually decreased after the early larval developmental stages, such as 1st- and 2nd-instar larvae (Fig 3a). We also examined *miR-3260* expression in the corpora allata, which synthesizes and secretes JH. *miR-3260* expression in the corpora allata of the 5th-instar larvae on the 4th day was significantly higher than that in the whole-body (Fig 3b).

**Fig 3:**
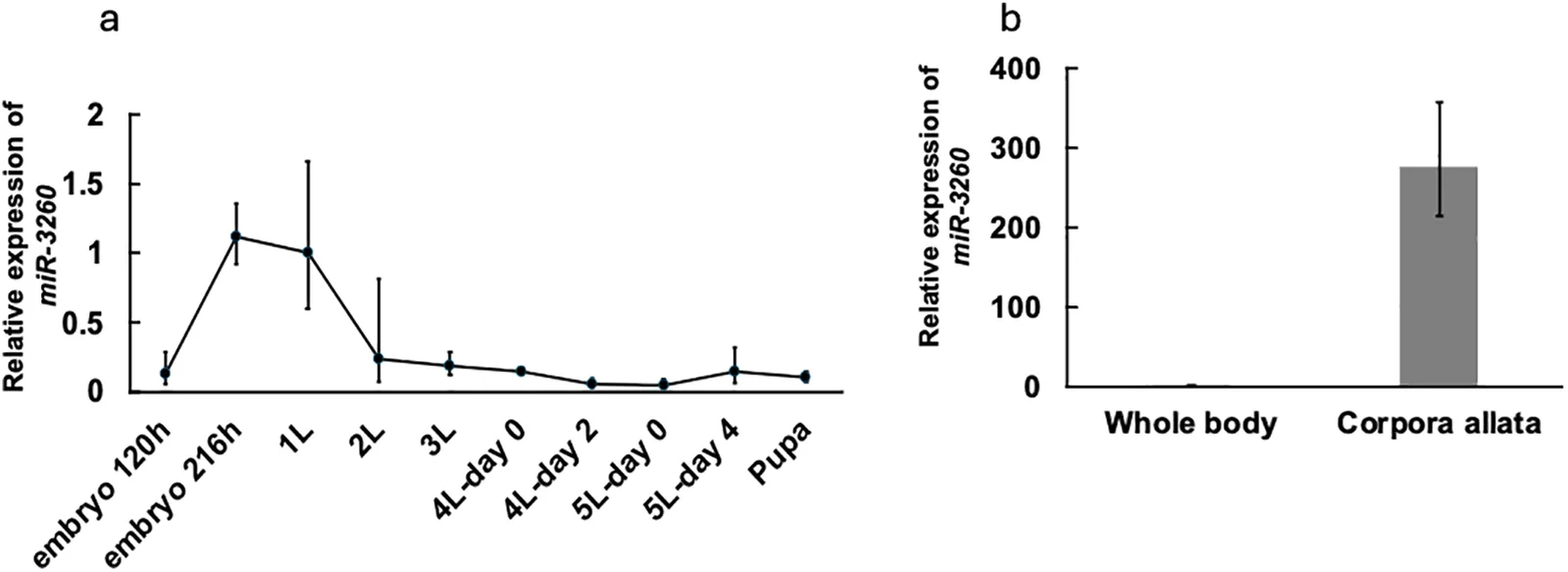
*miR-3260* expression at different developmental stages and in the corpora allata. (a) *miR-3260* expression during development. Embryo 120 h and 216 h indicate 120 and 216 h after oviposition, respectively; 1L, 2L, and 3L indicate 1st-, 2nd-, and 3rd-instar larvae, respectively; 4L-day 0 and 4L-day 2 indicate days 0 day and 2 of the 4th-instar larvae, respectively; 5L-day 0 and 5L-day 2 indicate days 0 day and 2 of the 5th-instar larvae, respectively. Whole-body miRNA expression levels are shown as RQ values, representing relative expression levels normalized against 1st-instar larvae samples (RQ = 1). Error bars represent RQ minimum and maximum levels based on standard deviation. U6 was used as an endogenous control. All sample sets were assayed in triplicate. (b) *miR-3260* expression in the corpora allata. miRNA expression levels in the corpora allata are shown as RQ values, which represent the relative expression levels calculated by assuming the value for whole bodies of 4-day 5th-instar larval samples as 1. Error bars indicate RQ minimum and maximum levels based on the standard deviation. U6 was used as an endogenous control. All sample sets were tested in triplicate.

### *miR-3260* expression after JH treatment in BmN cells

We further explored the relationship between *miR-3260* and JH by treating BmN cells with JH and measuring *miR-3260* expression at 24 and 48 h. *miR-3260* expression increased ~2.5-fold compared with that in the control group at 24 h (Fig 4). *miR-3260* expression also increased ~6-fold compared with that in the control group at 48 h (Fig 4). Thus, *miR-3260* responded to JH treatment in BmN cells. Since the *miR-3260* expression in the corpora allata related to JH biosynthesis and its upregulation with JH treatment, the subsequent experiment focused on *JHAMT* and *JHBP*.

**Fig 4:**
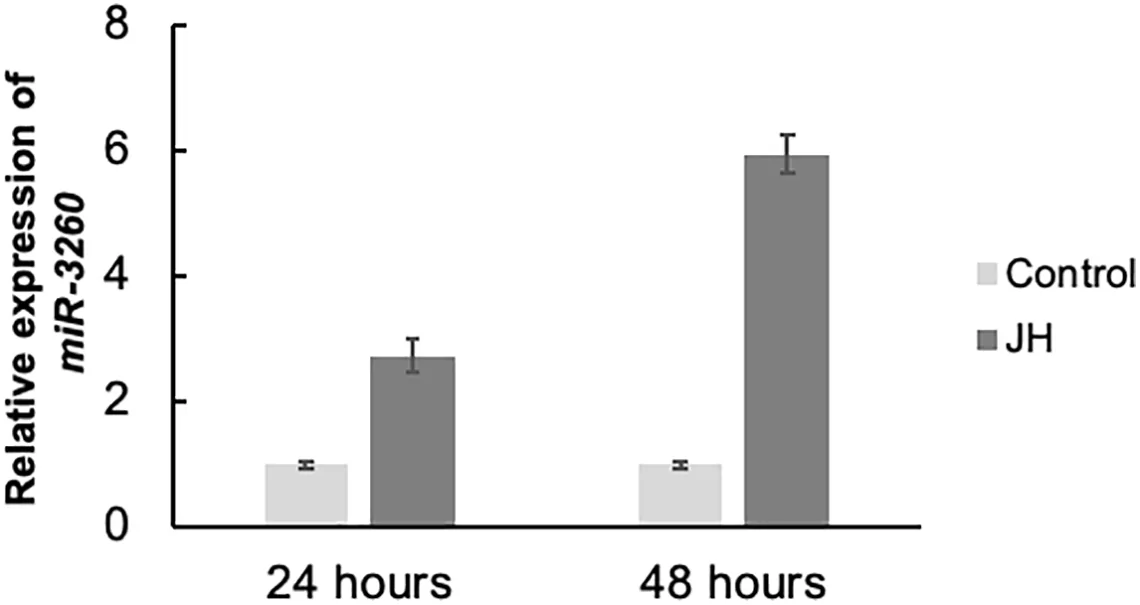
*miR-3260* expression in BmN cells treated with juvenile hormone for 24 or 48 h. miRNA expression levels in the BmN cells are shown as RQ values, which indicate relative expression levels calculated assuming the value for the control samples as 1. The white-gray column shows the control and the dark-gray column JH treatment. Error bars represent RQ minimum and maximum levels based on the standard deviation. U6 was used as an endogenous control. All sample sets were tested in triplicate.

### JH-related gene expression at various developmental stages

We measured *JHAMT* and *JHBP* mRNA expression from the embryonic to the pupal developmental stages. *JHAMT* mRNA expression was highest in the embryo at 120 h after oviposition (Fig 5a) whereas *JHBP* mRNA expression was highest at 216 h after oviposition, followed by a sharp decline at early larval stages (Fig 5b).

**Fig 5:**
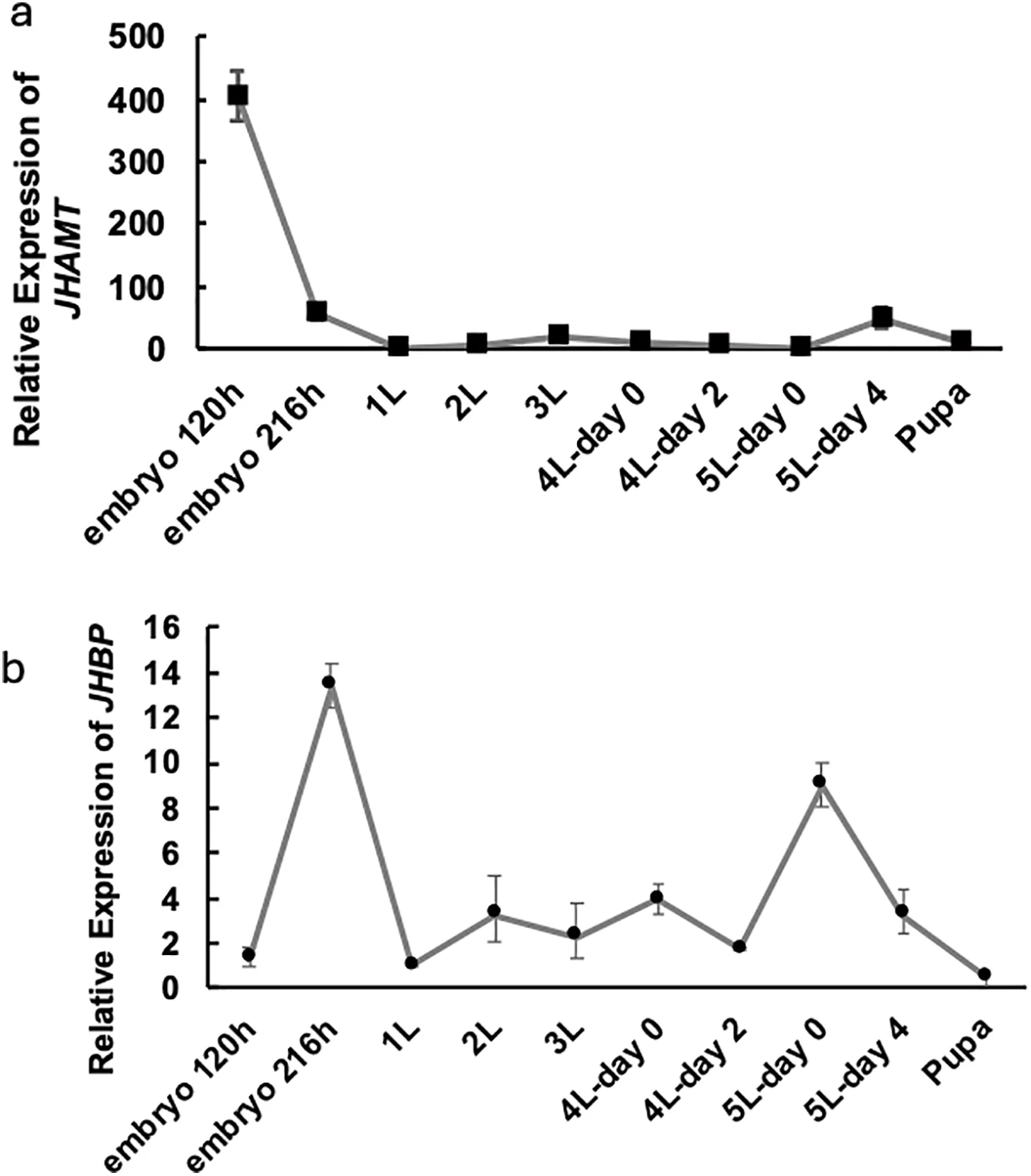
*JHAMT* and *JHBP* mRNA expression at various developmental stages. (a) *JHAMT* mRNA expression, (b) *JHBP* mRNA expression. Embryo 120 h indicates 120 h after oviposition; Embryo 216 h indicates 216 h after oviposition; 1L indicates 1st-instar larva; 2L indicates 2nd-instar larva; 3L indicates 3rd^.^-instar larva; 4L-day 0 indicates day 0 of the 4th-instar larva; 4L-day 2 indicates day 2 of the 4th-instar larva; 5L-day 0 indicates day 0 of the 5th-instar larva. 5L-day 2 indicates day 2 of the 5th-instar larva. mRNA expression levels in whole bodies are shown as RQ values, which represent the relative expression levels calculated by assuming the value for 1st-instar larval samples as 1. Error bars indicate RQ minimum and maximum levels based on the standard deviation. *Rp49* was used as an endogenous control.

### Phenotype obtained after *miR-3260* mimic or inhibitor injection

We assessed the phenotype and underlying relationship between *miR-3260* and the of *JHAMT* and *JHBP* expression during embryogenesis by injecting a *miR-3260* mimic or inhibitor. *miR-3260*, *JHAMT*, and *JHBP* expression suggested a possible role for embryonic and early larval development. First, we tested our injection methods using a single-guide RNA targeting *BmBLOS2*. Genome editing for *BmBLOS2* causes a translucent larval skin phenotype [41]. There was no significant difference in hatching rates compared with the control group (*P* = 0.201; S2 Fig in S1 File). Therefore, we concluded that our injection method would work for the mimic/inhibitor. Injecting the *miR-3260* mimic (*P* = 0.3221) or inhibitor (*P* = 0.1071) at larval hatching did not affect hatching rate or larval appearance compared with the control (Fig 6a). Subsequently, *miR-3260* mimic was injected into eggs, and *JHAMT* and *JHBP* mRNA expression levels measured after development to the 2nd-instar larval stage (Fig 6b and c). *JHAMT* mRNA was markedly upregulated following injection of either *miR-3260* mimic or inhibitor into eggs (Fig 6b). In contrast, *JHBP* mRNA expression slightly decreased after *miR-3260* mimic injection but remained unchanged with inhibitor injection (Fig 6c).

**Fig 6:**
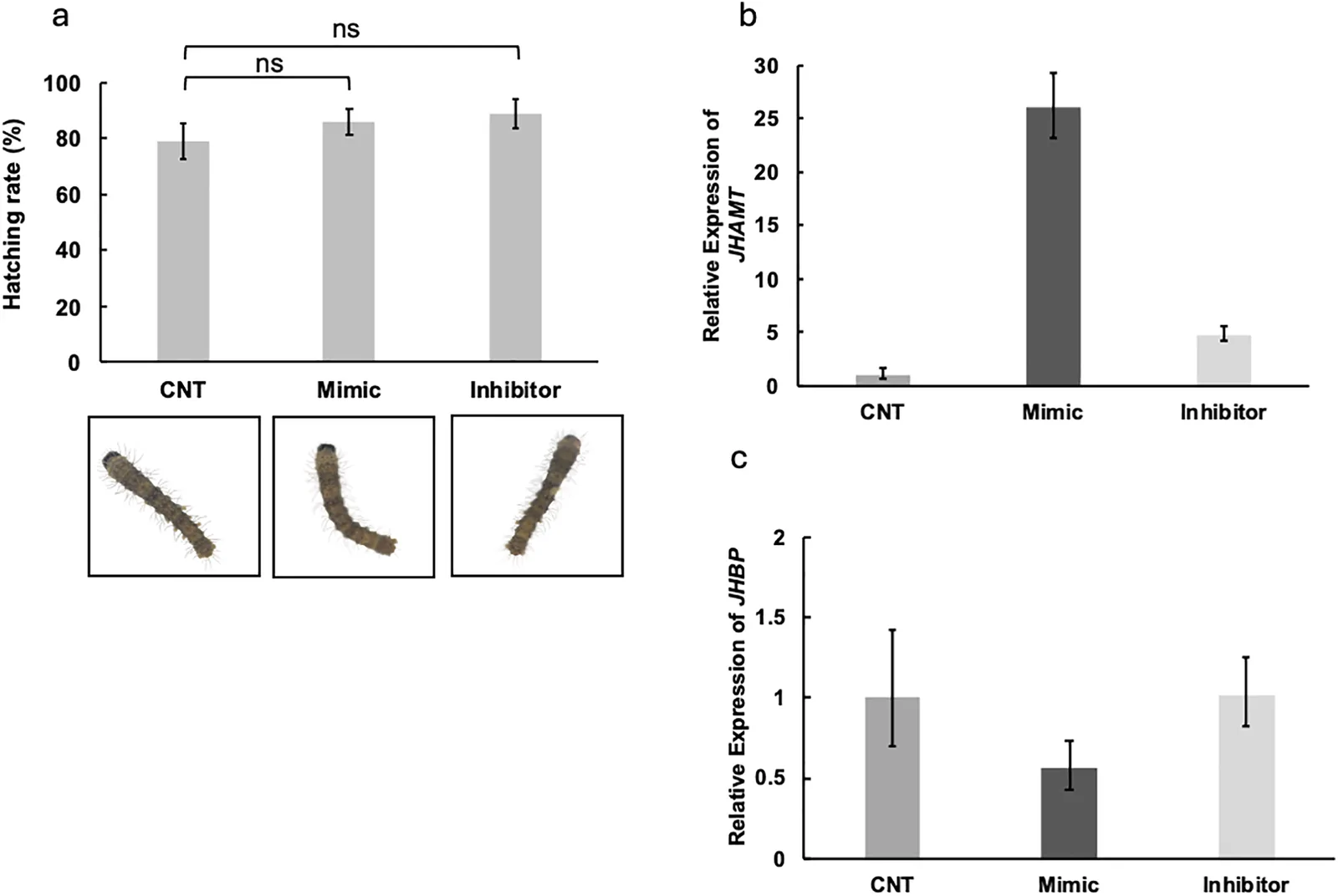
Effects of a *miR-3260* mimic or inhibitor. (a) Top, hatching rate; bottom, phenotype of 1st-instar larva. ns indicates non significant. (b) *JHAMT* mRNA expression after injection of the *miR-3260* mimic or inhibitor. (c) *JHBP* mRNA expression after injection of the *miR-3260* mimic or inhibitor; mRNA expression levels in each sample are shown as RQ values, which represent the relative expression levels calculated assuming the value for the control samples as 1. Error bars represent RQ minimum and maximum levels based on the standard deviation. Rp49 was used as an endogenous control.

### Dicing activity on pre-*miR-3260* and pre-m*miR-3260*

The stem‒loop structure in the miRNA precursor [9] is processed by Dicer [10,11]. This process plays a crucial role in the biosynthesis of mature miRNAs [12]. To examine how alterations in the stem‒loop structure affect production of the mature miRNA, we performed a dicing assay using cell-free crude extracts prepared from BmN cells and fat bodies. Using a 50-nt perfectly matched double-stranded RNA (dsRNA) with blunt ends as substrate, a 20-nt RNA cleaved by *BmDicer-2* was detected (Fig 7a and d, gray asterisks), as reported previously [39]. The wild-type *bmo-let7* precursor (*pre-bmo-let7*) was cleaved into a 22 nt fragment (miRbase, MIMAT0015221) by putative *BmDicer-1* activity (Fig 7b and d, red asterisks), but the mutant *bmo-let7* precursor *(pre-bmo-*m*let7*) modified to reduce susceptibility to Dicer cleavage was not cleaved compared to the *bmo-let7* precursor (Fig 7d, green asterisks). Accordingly, we concluded that our experimental system is valid. According to the information in miRbase (MIMAT0015444), mature *miR-3260* is 24 nt. Although the intensity of the ^32^P-labeled *pre-miR-3260* substrate band appeared lower than that of *pre-miR-3260* (comparing the wild-type FB and CC lanes with the corresponding mutant lanes), neither precursor exhibited cleavage at the expected ~24-nt positions (Fig 7c and d).

**Fig 7:**
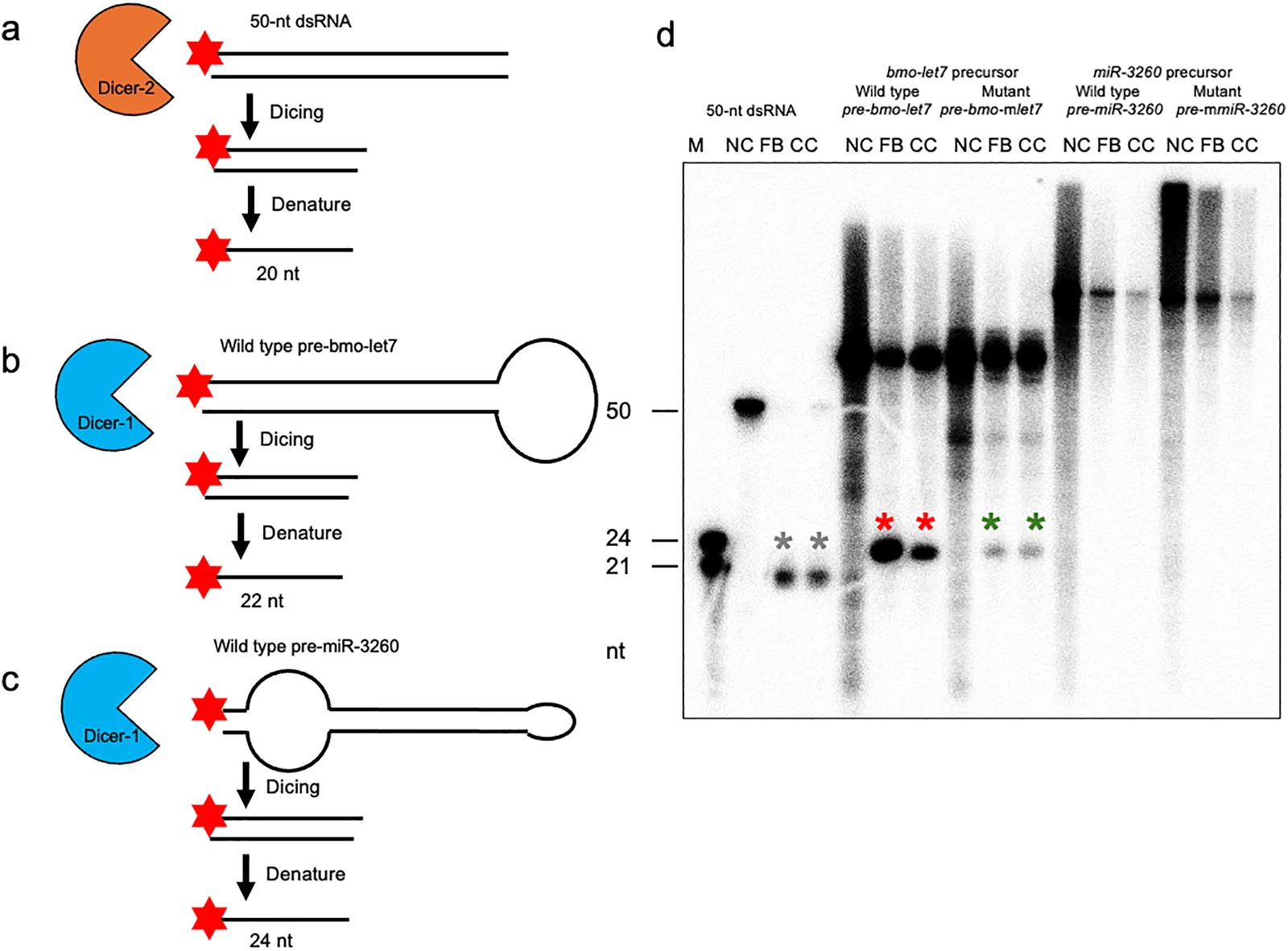
Comparison of dicing activity between *pre-miR-3260* and *pre-*m*miR-3260* Schematic diagram of the dicing assay: (a) 50-nt dsRNA, (b) wild-type bmo-let7 precursor, and (c) wild-type miR-3260 precursor. The red star indicates ^32^P. (d) A 50-nt dsRNA was used as a control. The bmo-let7 precursor (*pre-bmo-let7*) was used as a positive control. The mutant *bmo-let7* precursor (*pre-bmo-*m*let7*) was used as negative control. Wild-type and mutant *miR-3260* precursor. Lane M, RNA size markers of 50, 24, and 21 nt; NC, negative control (no-enzyme fraction); FB, fat body; CC, cultured BmN cells. Gray asterisks indicate products diced by *BmDicer-2*, whereas red or green asterisks indicate those diced by *BmDicer-1*. Original autoradiography images were provided in S3 Fig in S1 File.

## Discussion

In this study, we found that m*miR-3260* lacked some nucleotide sequences on the predicted loop structure of the o-751 silkworm strain. Since the same sequence is conserved in the *op*/*op*, + /*op*, and +/+ in the o-751 silkworm strain, this mutation is unlikely a direct cause of *op*. Therefore, although m*miR-3260* is unlikely to cause the *op* phenotype, its biological role should be considered independently of the known causative mechanisms of *op*.

While searching for *miR-3260* binding partners, we identified eight candidates. *miR-3260*, located at 9795152–9795269 on chromosome 23, had upstream JHRE sequences. The JHRE is a cis-regulatory element that mediates JH-induced transcriptional activation. Upon JH stimulation, the Methoprene-tolerant (Met)/Taiman (Tai) receptor complex directly binds to JHRE to initiate target gene expression [40]. The JHRE core typically features a highly conserved E-box motif (cacgtg) which serves as a critical docking site for these bHLH transcription factors [40].

The binding affinity of miRNA to its target mRNA is indicated by the mfe, typically reported to occur ≤ −20.0 kcal/mol; the lower the free energy value, the stronger the binding [37,38,42,43]. The mfe for *JHAMT* and *miR-3260* was −22.6, and that for *JHBP* and *miR-3260* was −24.1. Thus, *miR-3260* may bind to these mRNAs. Although *JHBP* was not detected in the fat body RNA-seq dataset, as its transcripts were absent from that tissue, it was subsequently selected for further analysis because it is located near *miR-3260* on chromosome 23, is part of the JH-related gene cluster, and is predicted to be a potential target of *miR-3260*. Furthermore, RT-qPCR analyses of whole-body samples revealed *JHBP* expression during embryonic and larval development, suggesting that *JHBP*’s absence from the RNA-seq dataset likely reflects tissue-specific expression.

*miR-3260* expression was markedly upregulated from 216 h after oviposition to the first-instar larval stage, coinciding with new JH synthesis [44]. Additionally, it was significantly higher in the corpora allata of 5th-instar larvae on day 4, and JH treatment of BmN cells also raised *miR-3260* expression levels. Furthermore, analyzing *miR-3260*’s upstream region revealed the presence of an E-box motif, indicating potential JH-responsive transcriptional regulation. Based on these observations, we hypothesized that *miR-3260* is involved in JH signaling. Target prediction analysis identified eight candidate genes potentially regulated by *miR-3260*, including *JHAMT* and *JHBP*, which are crucial for JH biosynthesis and transport, respectively. Prior studies reported sustained *JHAMT* and *JHBP* mRNA expression after dorsal closure during embryogenesis [44].

*JHAMT* methylates farnesoic acid to produce the final product, JH [45]. *JHBP* is annotated as ce-0330 in the *B. mori* compound eye expressed sequence tag database [46]. Many JH-binding proteins are secreted into the hemolymph, where they bind to JH and transport it to the target cells [46]. However, JHBP’s amino acid sequence lacks secretion signals, suggesting a cytosolic role [46]. Notably, *miR-3260* reached peak expression from 216 h after oviposition through the 1st-instar larval stage, whereas *JHBP* expression declined significantly in 1st-instar larvae. During embryonic development, the JH titer increases from 96 h and remains elevated after hatching [44]. Consequently, we analyzed the relationships among *miR-3260*, *JHAMT*, and *JHBP* across the eight predicted candidates.

Next, to investigate phenotypes and relationships among *miR-3260*, *JHAMT*, and *JHBP* during embryogenesis, we injected an *miR-3260* mimic and inhibitor into *B. mori* eggs. The hatching rates did not differ among groups, and the *miR-3260* mimic or inhibitor did not affect the phenotype of 1st-instar larvae. JH‑dependent transcriptional responses may be robust and reproducible at larval stages, while embryonic stages may be strongly influenced by maternally deposited transcripts that confound gene regulation interpretation [47]. In addition, functional manipulation of JH signaling, including use of JH analogs and RNAi-based approaches, has been widely performed in larval stages, where delivery and phenotypic assessment are experimentally tractable [48,49]. Thus, we evaluated *JHAMT* and *JHBP* mRNA expression in 2nd-instar larvae. Injection of the *miR-3260* mimic significantly upregulated *JHAMT* mRNA expression while slightly downregulating *JHBP*. Similarly, the *miR-3260* inhibitor upregulated *JHAMT* mRNA expression but did not affect *JHBP*. Therefore, the present outcomes did not align with the predicted regulatory functions of either *miR-3260* mimic or inhibitor. The observed phenomenon following mimic administration may represent a RISC saturation artifact [50] caused by an overabundance of nonfunctional RNA. Therefore, we propose that the effects of the *miR-3260* mimic and inhibitor on *JHAMT* and *JHBP* expression likely arise from off-target effects under our experimental conditions, rather than from direct regulation by *miR-3260*.

The stem‒loop structure in the miRNA precursor plays a crucial role in processing mature miRNAs. m*miR-3260* lacked an 8-nt sequence in its predicted loop structure. Dicer recognizes the 3’ overhanging end structure of the substrate and measures the distance from that structure to the single-stranded RNA region, and the binding ability decreases when the stem structure is long or when the loop structure is small [51]. Dicer cleaves pre-miRNAs into duplexes comprising a guide strand and a star strand, with the guide strand incorporated into Argonaute (AGO) and the star strand getting subsequently degraded [52]. In our dicing assay, Dicer recognition relied on the 5’ phosphate site. If Dicer had cleaved the ^32^P-end–labeled 50-nt dsRNA from the opposite end, we would have detected a 30-nt fragment (Fig 7d). The sequence and structural features of pre-miRNA hairpins, such as the 5′-phosphate and the apical loop, are important for Dicer-1’s processing activity [39,53]. Further, the 3’ fragment of the dsRNA cleaved by Dicer from the precursor miRNA becomes mature *miR-3260*. Normally, the antisense strand (star strand) of a miRNA duplex is cleaved by AGO; however, AGO activity could not be detected in our dicing assay. Therefore, if Dicer-1 cleaves pre-*miR-3260* in this experimental system, a 24-nt ^32^P-end–labeled star strand RNA should be detected. However, we did not observe Dicer-mediated cleavage of either the wild-type or mutant *miR-3260* precursor.

Because the results obtained using *miR-3260* mimics and inhibitors may have been influenced by off-target effects, they should be interpreted with caution and do not permit definitive conclusions regarding *miR-3260* function. In addition, canonical miRNAs can regulate gene expression not only through mRNA degradation but also through translational repression. Therefore, the absence of clear changes in transcript abundance does not completely exclude the possibility of regulatory effects at the protein level. However, because neither the wild-type nor mutant precursor was processed by Dicer in our experiments, the evidence supporting a conventional miRNA function of *miR-3260* remains limited. Taken together, these findings raise the possibility that miR-3260 represents a JH-responsive noncoding RNA or an atypical RNA distinct from canonical miRNAs.

It remains unclear why *miR-3260* responds to JH and is highly expressed in the corpus allatum. Although the presence of upstream E-box-like sequences suggests possible regulation by components of the JH signaling pathway, the molecular mechanisms underlying this response remain unresolved. Current experimental approaches are limited in their ability to clarify *miR-3260* function. Future technological advances may provide further mechanistic insights. Based on our results, the stem‒loop structure of *miR-3260* may be resistant to canonical Dicer cleavage, suggesting that both wild-type and mutant *miR-3260* may not act as conventional miRNAs.

## Conclusion

Our findings suggest that *miR-3260* is regulated by JH and is preferentially expressed in the corpus allatum, although the underlying mechanisms remain unclear. The presence of E-box-like sequences in the upstream region of *miR-3260* raises the possibility that components of the JH signaling pathway may contribute to its regulation. However, the functional significance of these sequences remains to be experimentally determined. In addition, the absence of detectable Dicer-mediated processing suggests that *miR-3260* may not function as a conventional miRNA. Further research focusing on its transcriptional regulation, biogenesis, and molecular interactions is necessary to clarify its role in JH signaling. Collectively, *miR-3260* may represent a novel JH-responsive atypical RNA distinct from canonical miRNAs.

## Supporting information

S1 File Supporting figures (S1-S3 Figs), supporting tables (S1-S5 Tables), and methods for RNA-seq analysis. (PDF)
