# Supplementary material for: Identification of an atypical microRNA with sequence variation in the silkworm Bombyx mori
Source: PLoS One. 2026 Oct 6;21(10):e0346656. doi: 10.1371/journal.pone.0346656 (PMC13641386; doi:10.1371/journal.pone.0346656)
Supplement: Supplementary file 1 [file pone.0346656.s001.pdf]

Supporting information

```
WT      AUGCAAGAUAGAAGAAGUAGAAUGAAAGAUAGUAAGAAUGAAAGAAAAUAAAUCCGCAAU
MT      AUGCAAGAUAGAAGAAGUAGAAUGCAAGAUAGCAAGAAUGCAAGAAAAUAGAU-------
        *****************************************************

WT      CUGGGGGUUUACUUUUCGGUUUAAUGUUCACUGUCUCUUGAUUACUUUCGUGAGUUGA
MT      -UGCGGGUUUACUUUUCGGUUUAAUGUUCACUGUCUCUUGAUUACUUUCGUGAGUUGA
         *********************************************************
```

S1 Fig. Alignment of wild type and mutant type of *miR-3260*. WT indicates wild type, and MT indicates mutant type. The multiple sequence alignment was performed using CLUSTAL O (1.2.4). The hyphens indicate a lack of nucleotide sequence. The black asterisks indicate the same nucleotide sequences between wild-type and mutant types of *miR-3260*. The gray asterisks indicate the different nucleotide sequences between wild-type and mutant types of *miR-3260*. The red asterisks indicate mature sequences.

a

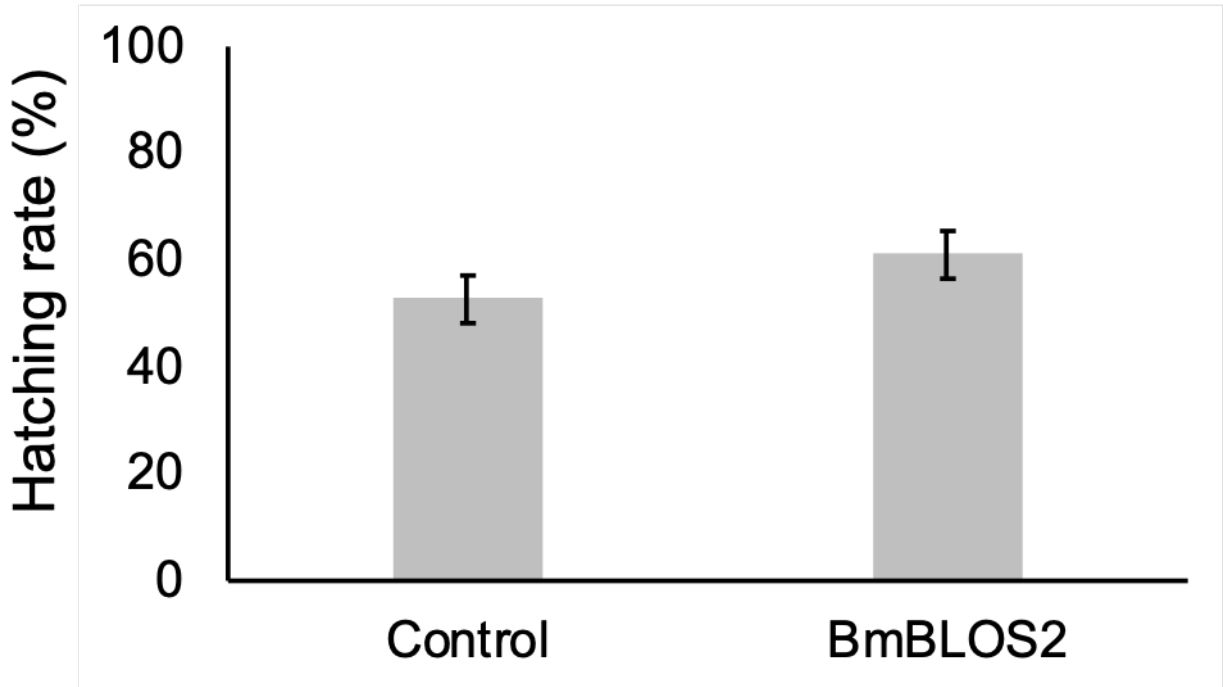

b

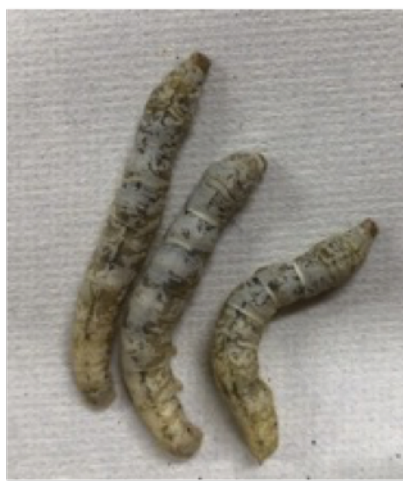

S2 Fig. Assessment of egg injection methods. BmBLOS2 single-guide RNA (sgRNA) was designed according to Daimon et al. (Develop. Growth Differ. (2014) 56, 14–25). A mixture of sgRNA and Cas9 protein was injected into eggs of the *Bombyx mori* N4 strain, and the hatching rate was evaluated.

(a) Hatching rate in each group. Control groups (n = 48 per group) were injected with water, whereas BmBLOS2 groups (n = 48 per group) were injected with the sgRNA–Cas9 protein mixture. TTEST, (P =0.201).

(b) BmBLOS2 phenotypes observed in fifth-instar larvae after genome editing of BmBLOS2.

The assessment of egg injection methods was performed in duplicate as biological replicates.

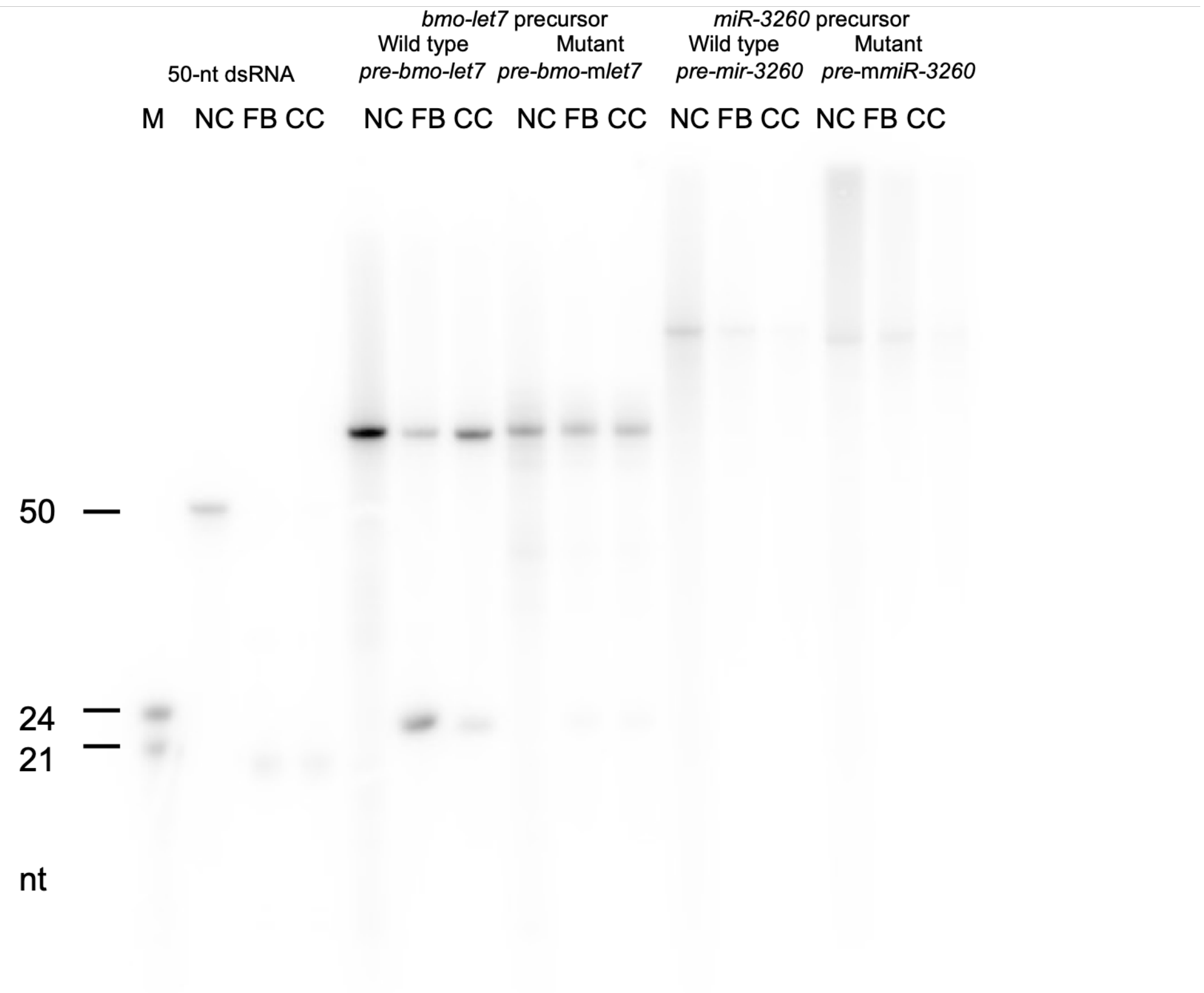

S3 Fig. Original autoradiography image for Fig. 7 d. Original autoradiography data were obtained by drying an RI-containing PAGE gel, exposing it to an imaging plate, and scanning the plate using a Typhoon FLA 7000 image analyzer and ImageQuant TL (GE Healthcare Japan). The original autoradiography image was processed using ImageQuant TL in High-contrast mode. Then, the original autoradiography image was cropped in Preview to remove the left and right margins.

S1 Table. Positional cloning of the *op* candidate gene.

Marker	Chromosome position	o75	C108	F_1 female	F_1 male	F_2*															
						batch 1							batch 2							batch 3	batch 4
						#2	#9	#15	#27	#33	#38	#40	#18	#29	#59	#61	#67	#68	#71	#9	#6
Bm_scaf22-4F/4R	8062853	A	B	A/B	A/B	A	A	A	A/B	A	A/B	A	A	A	A	A	A	A/B	A/B	A	A
Bm_scaf22-1F/1R	8991486	A	B	A/B	A/B	A	A	A	A	A	A	A	A	A	A	A	A	A	A/B	A	A
**Bm_scaf22-22F/22R**	**9598832**	A	B	A/B	A/B	A	A	A	A	A	A	A	A	A	A	A	A	A	A/B	A	A
Bm_scaf22-10F/10R	9687077	A	B	A/B	A/B	A	A	A	A	A	A	A	A	A	A	A	A	A	A	A	A
**Bm_scaf22-23F/23R**	**9916751**	A	B	A/B	A/B	A	A	A	A	A	A	A	A	A	A	A	A	A	A	A/B	A
Bm_scaf22-14F/14R	10377814	A	B	A/B	A/B	A	A	A/B	A	A	A	A/B	A/B	A/B	A/B	A	A/B	A	A	A/B	A/B
Ch23-2F/2R	11071323	A	B	A/B	A/B	A/B	A/B	A/B	A	A/B	A	A/B	A/B	A/B	A/B	A/B	A/B	A	A	A/B	A/B

'A' indicates o75 homozygous, 'B' indicates C108 homozygous, and 'A/B' indicates heterozygous genotype.

Heterozygous genotypes are shaded.

The bold letters indicate primer sets located most closely to *op* linked region.

The chromosome position responsible for *op* is the area enclosed by the square.

* In positional cloning, 256 F_2 individuals with the *op* phenotype were used.

S2 Table. Primers used for positional cloning.

Primer name	Forward	Reverse
Ch23-2	5’-AGTAACAACGCGCCTTATTGTCAAA-3’	5’-ATGTATAGCGAAGCGTAGCCGGTAT-3’
Bm_scaf22-1	5’-AGTGATAATTCAAGACCGGCACCTT-3’	5’-CTTTTCGGGATTATCTCGCTTTCAC-3’
Bm_scaf22-4	5’-TCACTTAATTTCTGAAGTTTGCACAAAAA-3’	5’-GATGCTCATCGGAGTCAAATCGTTA-3’
Bm_scaf22-10	5’-AAGCAATTATTCACAACACCGATGG-3’	5’-GTTTTGTTTGACCGAGTCTTTGTGG-3’
Bm_scaf22-14	5’-CTGTGCCTCTAGCGCTGATGATATT-3’	5’-TGTTTGCGAAAATAGCCATTAAACG-3’
Bm_scaf22-22	5’-AAGCCTTTGGAAAGATGTCGCTAGA-3’	5’-CTGAAACGATGCACACAATAGTTCG-3’
Bm_scaf22-23	5’-GGAATAGTTTGATCCCTGGGAAAGA-3’	5’-AAAGCGGATTTTGAAATGGTTACTGA-3’

S3 Table. Primers used for miRNA cloning and qRT–PCR.

Primer name	Forward	Reverse
M13	5’-TGTAAAACGACGGCCAG-3’	5’-CAGGAAACAGCTATGA-3’
Rp49	5’-CCAACATTGGTTACGGTTCCA-3’	5’-CATTGTGAACTAGGACCTTACGGAAT-3’
JHAMT	5’-TGCGCTGACGGTAGTGTTAC-3’	5’- AGCTCGTTCCTGGTCTCTGA-3’
JHBP	5’-AAGGGACACAAGAACGCAAC-3’	5’- TCTTTGCCATCCTCTGCTTT-3’

S4 Table. Nucleotide sequences used for the mimic and inhibitor of *B.mori miR-3260*.

**Product**	**Nucleotide sequence**
bmo-miR-3260 ID MC18121 mimic (antisense)	5’-UGUCUCUUGAUUACUUUCGUGAGU-3’
bmo-miR-3260 ID MC18121 mimic (sense)	5’-UCACGAAAGUAAUCAAGAGACATT-3’
bmo-miR-3260 ID MH18121 inhibitor	5’-ACUCACGAAAGUAAUCAAGAGACA-3’

S5 Table. Nucleotide sequences used for the chemical synthesis of pre-miRNAs and dsRNA.

**Pre-miRNA**	**Nucleotide sequence**
pre-*miR-3260* (118 base)	5’-AUGCAAGAUAGAAGAAGUAGAAUGAAAGAUAGUAAGAAUGAAAGAAAAUAAAUCCGCAAUCUGGGGUUUACUUUUCGGUUUAAUGUUCACUGUCUCUUGAUUACUUUCGUGAGUUGA-3’
Pre-m*miR-3260* (110 base)	5’-AUGCAAGAUAGAAGAAGUAGAAUGCAAGAUAGCAAGAAUGCAAGAAAAUAGAUUGCGGGUUUACUUUUCGGUUUAAUGUUCACUGUCUCUUGAUUACUUUCGUGAGUUGA-3’

pre-*bmo-let-7* (68 base)	5'-UGAGGUAGUAGGUUGUAUAGUACGGAAAUACAACACAUAGGUGCGACUGUAUAGCCUGCUAACUUUCC-3'
pre-*bmo-*m*let-7* (69 base)	5'-UGAGGUAGUAGGUUGUAUAGUACGGAAAUACAACAUGUAUUUCCGUACUGUAUAGCCUGCUACCUUUCC-3'
50-nt dsRNA sense	5'-UACAAGCGAAUGAGUCAUUCAUCCUAAGUCCAACAUAGCGUUCGAUAAGA-3'
50-nt dsRNA antisense	5'-UCUUAUCGAACGCUAUGUUGGACUUAGGAUGAAUGACUCAUUCGCUUGUA-3'

The method for RNA-seq analysis

Total RNA was isolated from the fat body of day 3 fifth-instar larvae of the *B. mori* o751, *op* strain using a PureLink® RNA extraction kit (Thermo Fisher Scientific Inc.) according to the manufacturer's protocol. The quality of RNA was assessed using an Agilent Bio-analyzer 2100 (Agilent Technologies, Santa Clara, CA, USA). Paired-end sequencing cDNA libraries were constructed with 4 μg of total RNA from o751 *op* strain of the fat body samples (n = 3) with a Truseq RNA Sample Preparation Kit Set A (Illumina Inc., San Diego, CA, USA) according to the manufacturer's protocol. RNA-seq was performed using a HiSeq 2500 system (Illumina Inc.). The data quality of the fastq files was verified with the FastQC tool (Babraham Bioinformatics, http://www.bioinformatics.babraham.ac.uk/projects/fastqc/). The 44M paired-end reads (2 × 150 bp) were mapped to the reference *B. mori* genomes available in the Ensembl Genome database[1] using the Tophat program version 2.0.13 with default parameters[2]. BAM formatted files generated by Tophat were sorted and indexed using SAMtools[3] and then converted to Wiggle track format (WIG) files using the bam2wig software (https://github.com/MikeAxtell/bam2wig). This allowed us to visualize the density of reads mapped to the specific region of interest. Histograms of mapped reads were generated using the Spotfire Cloud software with TIBCO Spotfire's "Better World" program license (TIBCO Software, Inc., Palo Alto, CA, USA) (http://spotfire.tibco.com/better-world-donation-program/).

References

1 Kersey PJ, et al. Ensembl Genomes 2016: more genomes,more complexity. Nucleic Acids Research. 2015; 4: D574-80

2 Kim D, Pertea G, Trapnell C, Pimentel H, Kelley R, Salzberg SL. TopHat2: accurate alignment of transcriptomes in the presence of insertions, deletions and gene fusions. Genome Biology. 2013;14:R36.

3 Li H., et al. The Sequence alignment/map (SAM) format and SAMtools. Bioinformatics. 2009;25:2078-9.
